# A Surface Conformal Laser‐Assisted Alloying Reaction for 3D‐Printable Solid/Liquid Biphasic Conductors

**DOI:** 10.1002/smsc.202200089

**Published:** 2023-02-03

**Authors:** Jiyun Shim, Yeon Uk Kim, Young-Bin Kim, Seul Gi Ji, Yeon Ju Kim, Yejin Jo, Eun Jung Lee, Do-Gyeong Yuk, Su Yeon Lee, Sun Sook Lee, Sun-Kyung Kim, Hyung-Seok Kim, Jung Hwan Park, Sunho Jeong

**Affiliations:** ^1^ Department of Advanced Materials Engineering for Information and Electronics Integrated Education Institute for Frontier Science & Technology (BK21 Four) Kyung Hee University Yongin-si 17104 Republic of Korea; ^2^ Department of Mechanical Design Engineering Kumoh National Institute of Technology 61 Daehak-ro Gumi Gyeongbuk 39177 Republic of Korea; ^3^ Department of Applied Physics Kyung Hee University Yongin-si 17104 Republic of Korea; ^4^ Division of Advanced Materials Korea Research Institute of Chemical Technology Daejeon 34114 Republic of Korea; ^5^ Department of Aeronautics, Mechanical and Electronic Convergence Engineering Kumoh National Institute of Technology 61 Daehak-ro Gumi Gyeongbuk 39177 Republic of Korea; ^6^ KHU-KIST Department of Converging Science and Technology Kyung Hee University Seoul 02447 Republic of Korea

**Keywords:** 3D printing, biphasic conductors, hierarchical particles, laser-assisted alloying, metallic inks

## Abstract

Recently, electronics research has made major advances toward a new platform technology facilitating form factor‐free devices. 3D printing techniques have attracted significant attention in the context of fabricating arbitrarily shaped circuits. Herein, a 3D‐printable metallic ink comprising multidimensional eutectic gallium indium (EGaIn)/Ag hierarchical particles is proposed to fabricate arbitrarily designable solid/liquid biphasic conductors that can be inherently self‐healed/chip bonded and do not suffer from liquid flood out due to their liquid and solid nature, respectively. The EGaIn/Ag hierarchical particles are designed to have plasmonic optical absorption at the visible green–red wavelength regime, which is elucidated by an optical simulation study, and also enable the direct transfer of thermal energy, generated in the vicinity of the Ag nanoparticles, to the surface of the EGaIn particles. The 3D surface conformal green laser irradiation process activates the evolution of the biphasic conductive layer from the as‐printed insulating particulate one. The chemical/physical evolution is elucidated along with a photothermal simulation study for clarifying the suppression of undesirable side reactions. It is demonstrated that the biphasic conductors formed by successive 3D printing and the surface conformal green laser irradiation process exhibit electrical properties that have thus far been unexplored in solid metallic conductors.

## Graphical abstract

Hierarchical particles comprising liquid metal particles and silver nanoparticles are designed to trigger photothermally activated interfacial alloying reactions. By subsequent direct ink writing (DIW) and green laser irradiation processes, biphasic conductors are generated on top of the 3D structure with characteristic properties that are not obtainable in conventional solid metallic features.

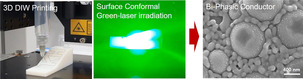

## 1 Introduction

Electronics research has moved toward a new platform technology that can facilitate an on‐demand design of form factor‐free devices, with the advent of the era of “internet of things” in which arbitrarily‐shaped daily objects are connected wirelessly with highly integrated ubiquitous systems.^[^ ^1^, ^2^, ^3^, ^4^ ^]^ Among recent advancements in processing techniques, the 3D printing process, which can deposit functional materials at desirable positions, has gathered tremendous attention in various applications.^[^ ^1^, ^2^, ^3^, ^4^ ^]^ In particular, there has been a growing demand in exploiting highly functioning 3D‐printable conductive materials.^[^ ^5^, ^6^, ^7^, ^8^, ^9^ ^]^ 3D conductive features are indispensably required for interconnecting active/passive components in integrated electronics systems. To date, a major concern has been focused on how to improve the electrical conductivity in printed conductor layers. In most previous studies, silver (Ag), copper (Cu), and nickel (Ni) solid nanoparticles have been explored as primary substances of building up 3D‐printed metallic structures.^[^ ^5^, ^6^, ^7^, ^8^, ^9^ ^]^ Recently, liquid metals with moderate electrical conductivity have been suggested as a viable candidate for realizing electrical/mechanical properties that cannot be satisfied in solid metal structures.^[^ ^10^ ^]^

Representative liquid metals are gallium‐based ones exhibiting low melting point even below room temperature. The gallium–indium alloys with eutectic melting points (EGaIn) have distinctive advantages of high conductivity, low toxicity, sufficient fluidity, and low vapor pressure.^[^ ^11^, ^12^ ^]^ Notably, the presence of an intrinsic inorganic skin layer does decrease the surface tension of liquid metals and also provides structural rigidity. It enables the fabrication of 3D liquid structures even in multistacked geometries.^[^ ^13^ ^]^ However, when there is enough external stress to collapse the ultrathin skin layer, the liquid metal cores spill out of the preformed structures and an uncontrollable outward flow results in an undesirable electrical disconnection. As an alternative method, the liquid metals can be mixed with polymers with the addition of good solvents. However, liquid metal inclusions are surface oxidized inside hybrid composites, which gives rise to an electrically insulating property.^[^ ^14^ ^]^ By deliberately imposing a compressive pressure in a specific trajectory, patterned conductive pathways can be generated in the hybrid composites,^[^ ^15^ ^]^ but there is still a critical problem in that the unwanted region is also activated in subsequent processing steps.^[^ ^16^ ^]^

As a new concept, biphasic conductors comprising solid and liquid metals have been suggested for acquiring both advantages from the solid and liquid metallic phases.^[^ ^17^, ^18^, ^19^, ^20^ ^]^ In case solid metal particles are mixed as a conductive additive with liquid EGaIn, the alloy reactions are triggered at the interface of solid metal particles and liquid EGaIn, which enables the formation of biphasic conductive materials. Lopes et al suggested that Ag flakes can be incorporated as a metallic additive for triggering alloying reactions at room temperature.^[^ ^18^ ^]^ It has been reported that Cu and Ni particles can take part in alloying reactions after additional chemical etching treatments for removing native oxide layers present on the surface of the Cu and Ni particles.^[^ ^19^, ^20^ ^]^ However, the chemical origin is still unclear for the intermetallic alloying reactions occurring even at room temperature without provision of thermal energy, and more importantly, 3D‐printable biphasic conductor materials and their potential advantages have not been reported yet.

Herein, we designed hierarchical multidimensional particles in which EGaIn micrometer‐sized droplets are surrounded sequentially with a gallium‐based oxide skin layer and Ag nanoparticles. The hierarchical particles (HPs) were formulated into a 3D‐printable fluid, and the surface conformal green‐laser irradiation process was carried out along the surface topology of 3D‐printed patterns, activating the laser‐assisted instantaneous intermetallic alloying reactions. Distinctively, the Ag nanoparticles were synthesized to have plasmonic absorption at a wavelength of 532 nm (the wavelength of a green laser). This effectively boosts the photothermal alloying reactions at the localized heterointerface between the Ag nanoparticles and EGaIn droplets. Such local heating enables the formation of the biphasic features in which the liquid EGaIn phase is captured by the outer indium–silver alloy phases without any thermal damage on the underlying substrates. It is demonstrated that the 3D‐printed biphasic metallic structure inherently self‐heals and chip bonds and does not suffer from liquid flood out of the printed patterns due to its liquid and solid nature, respectively, exhibiting electrical properties that have not been achievable in conventional solid metallic conductors.

## 2 Results and Discussion

### 2.1 Synthesis of Multidimensional EGaIn/Ag HPs

**Scheme 1:**
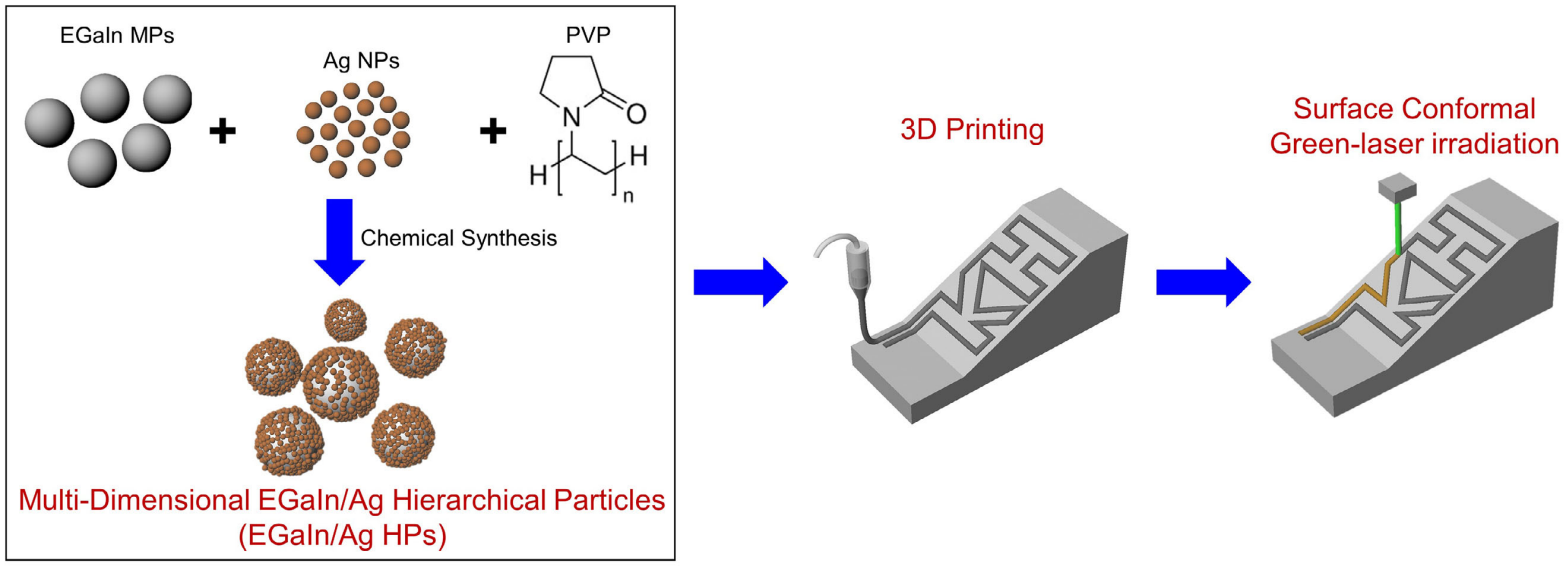
A schematic showing the successive procedures of fabricating 3D biphasic metallic structures by 3D printing and surface conformal green laser irradiation processes.

**Scheme** **1** shows the successive procedure for creating the 3D biphasic metallic structure by the 3D printing process and consecutive surface conformal green‐laser irradiation process in air. The multidimensional EGaIn/Ag HPs (EGaIn/Ag HPs) were synthesized by decorating the EGaIn micrometer‐sized particles (EGaIn MPs) with the Ag nanoparticles (Ag NPs). The dimension of Ag NPs was optimized to have a high absorption coefficient at a wavelength of 532 nm. The EGaIn/Ag HPs were formulated into highly viscoelastic ink appropriate to the 3D printing process by being mixed with a polymeric dispersant and a small amount of solvent. The 3D‐printed features undergo instantaneous alloying reactions for generating biphasic metallic structures by the surface conformal green‐laser irradiation process.

At first, we synthesized the Ag NPs using a polyol synthetic method. The silver nitrate was dissolved in ethylene glycol. Poly(vinylpyrrolidone) (PVP) was added, acting as both a capping molecule and a reducing agent. The compositions of PVP were 0.03, 0.01, and 0.007 m in the reaction solutions for Ag‐#1, Ag‐#2, and Ag‐#3 NPs, respectively. Details on the chemical compositions are shown in Table S1, Supporting Information. The Ag ions were reduced chemically by accepting electrons from the reducing agent at an elevated temperature of 120 °C. The scanning electron microscopy (SEM) images and size distribution histograms for Ag‐#1, Ag‐#2, and Ag‐#3 NPs are shown in Figure S1, S2, Supporting Information. The values in D50 and D90 for the Ag‐#1 NPs were 43.1 and 55.8 nm, respectively. Compared with those of the Ag‐#1 NPs, the values in D50 and D90 for the Ag‐#2 and Ag‐#3 NPs increased to 46.5 and 64.3 nm and 70.1 and 87.8 nm, respectively. By diminishing the concentration of the PVP in the reaction solution, the average diameter of the Ag NPs increased with an evolution of a wider particle size distribution. This tendency is attributed to the thermally driven random Brownian motion of the Ag nuclei during the growth reaction. Growth reactions proceed by additional adsorption of newly reduced Ag atoms toward the preformed nuclei and subsequent intercollisions between neighboring nuclei. The collision events are mitigated by the capping molecules surrounding the Ag nuclei; thus, Ag NPs become bigger by more frequent collision events when an insufficient amount of capping molecules are present.

**Figure 1:**
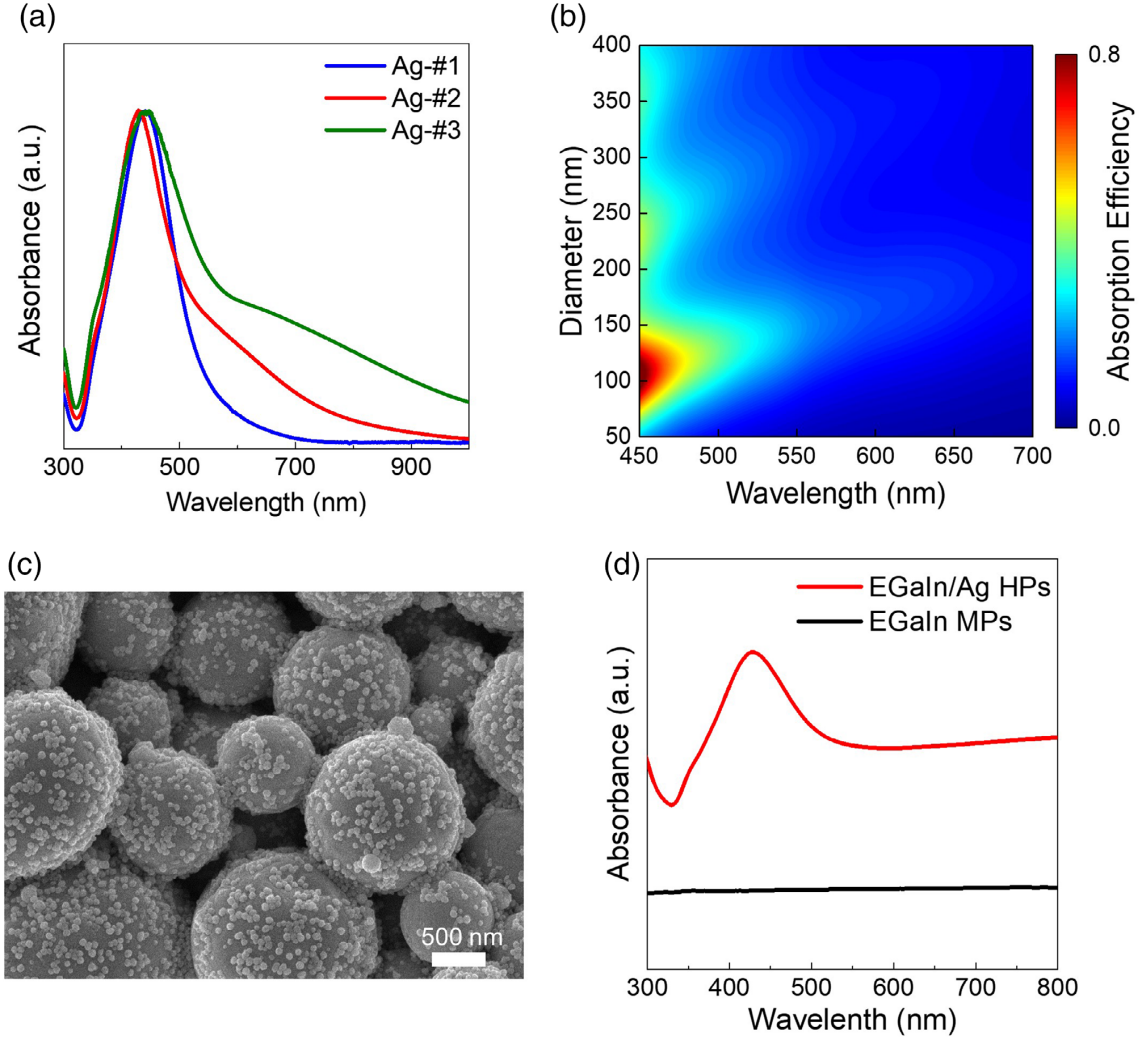
a) UV–vis spectra for Ag NPs synthesized in this study. b) Simulated absorption efficiency spectra (450–700 nm) of individual Ag NPs with various diameters. c) SEM image of EGaIn/Ag HPs synthesized in this study. d) UV–vis spectra of EGaIn MPs and EGaIn/Ag HPs.

According to the UV–vis spectra, the presence of larger nanoparticles in Ag‐#3 NPs leads to an absorption at a longer wavelength compared with the case of the Ag‐#1 NPs (**Figure** **1**a).^[^ ^21^ ^]^ This size‐dependent absorption characteristic was verified by optical simulations on the Ag NPs (Figure 1b). For these simulations, individual Ag NPs with various diameters (50–400 nm) were considered, and their scattering efficiencies were obtained in the visible spectrum (450–700 nm). The simulated results covey two key messages related to the plasmonic absorption of metal NPs. First, multiple scattering/absorption modes emerge at discrete wavelengths for a given Ag NP. Second, each mode progressively shifts to longer wavelengths by increasing the diameter of the Ag NPs. This behavior is consistent with the pronounced absorption of the larger Ag NPs at the green–red wavelengths (Figure 1a). A significant redshift of the plasmonic wavelength also evolves depending on the type of chemical bonding and the length of the organic capping ligand.^[^ ^22^, ^23^ ^]^ For the Ag NPs, redshift approaching a value of ≈40 nm was observed with the increase, from 3 to 15, of the number of carbons in the capping ligand,^[^ ^22^ ^]^ and redshift with a value of ≈40 nm occurs for capping ligands with a higher electron affinity (i.e., higher chemical binding energy).^[^ ^23^ ^]^ The PVP incorporated as a capping agent in this study has a long‐chain molecular structure and interacts with the metal atoms through strong coordination bonding by lone pairs in the pyrrolidone ring. In this regard, it can be speculated that the wavelength of plasmonic absorption for the Ag NPs is redshifted compared with the simulated one, a trend of which is more strengthened with the presence of larger Ag NPs. These results suggest that the Ag‐#3 NPs are more beneficial in absorbing the photons implanted from the green laser irradiation process and in activating designated biphasic alloying reactions.

The optical energy absorbed in the Ag NPs are transformed into thermal energy by the synchronized vibrations of the atoms positioned in the crystalline lattice sites, which is consumed by the triggered endothermic alloying reactions.^[^ ^24^, ^25^ ^]^ To build efficiently biphasic conductors, the thermal energy should be consumed entirely at the heterointerface between the solid Ag NPs and liquid EGaIn MPs. Highly efficient alloying reactions are accomplished only by a complete transfer of the thermal energy from the Ag NPs to the EGaIn MPs. Note that laser‐assisted alloying reactions are complete in an extremely short timescale of below 1 sec (e.g., suppose an irradiation process at a scan speed of 100 mm s^−1^ for a 10 cm‐long sample). Thus, a sophisticated design is important on the solid/liquid heterointerface for inducing the alloying reactions without a significant loss of thermal energy toward the outer surroundings. In case both Ag NPs and EGaIn MPs are mixed simply by conventional ink formulation methods, a small part of the Ag NPs establishes intact contacts with the EGaIn MPs, and consequently, most of the thermal energy vanishes and is not transferred to the heterointerface with the EGaIn MPs. In this study, we designed the EGaIn/Ag HPs by placing directly all the Ag NPs in a monolayered stacked sequence on the surface of the EGaIn MPs (Figure 1c). The EGaIn MPs were synthesized with the spontaneous formation of a native oxide skin layer surrounding the liquid droplets (Figure S3, Supporting Information).^[^ ^26^, ^27^, ^28^ ^]^ The hierarchical multidimensional particles were subsequently synthesized by decorating the EGaIn MPs with Ag NPs through the binder role of the additional PVP that anchors oxide‐passivated EGaIn MPs and Ag NPs. The monomer units of PVP can bond with hydroxyl groups present on the surface of oxide skin layer through chemical interactions by oxygen atoms of the carbonyl group and nitrogen atoms of the pyrrolidone ring.^[^ ^29^ ^]^ Figure 1d shows the UV–vis spectra of the EGaIn MPs and EGaIn/Ag HPs. It was observed that the EGaIn/Ag HPs have a much higher absorption property at a wavelength of 532 nm due to the presence of the Ag‐#3 NPs on top of the EGaIn MPs.

### 2.2 Green‐Laser‐Assisted Alloying Reaction for Creating Biphasic Conductors

**Figure 2:**
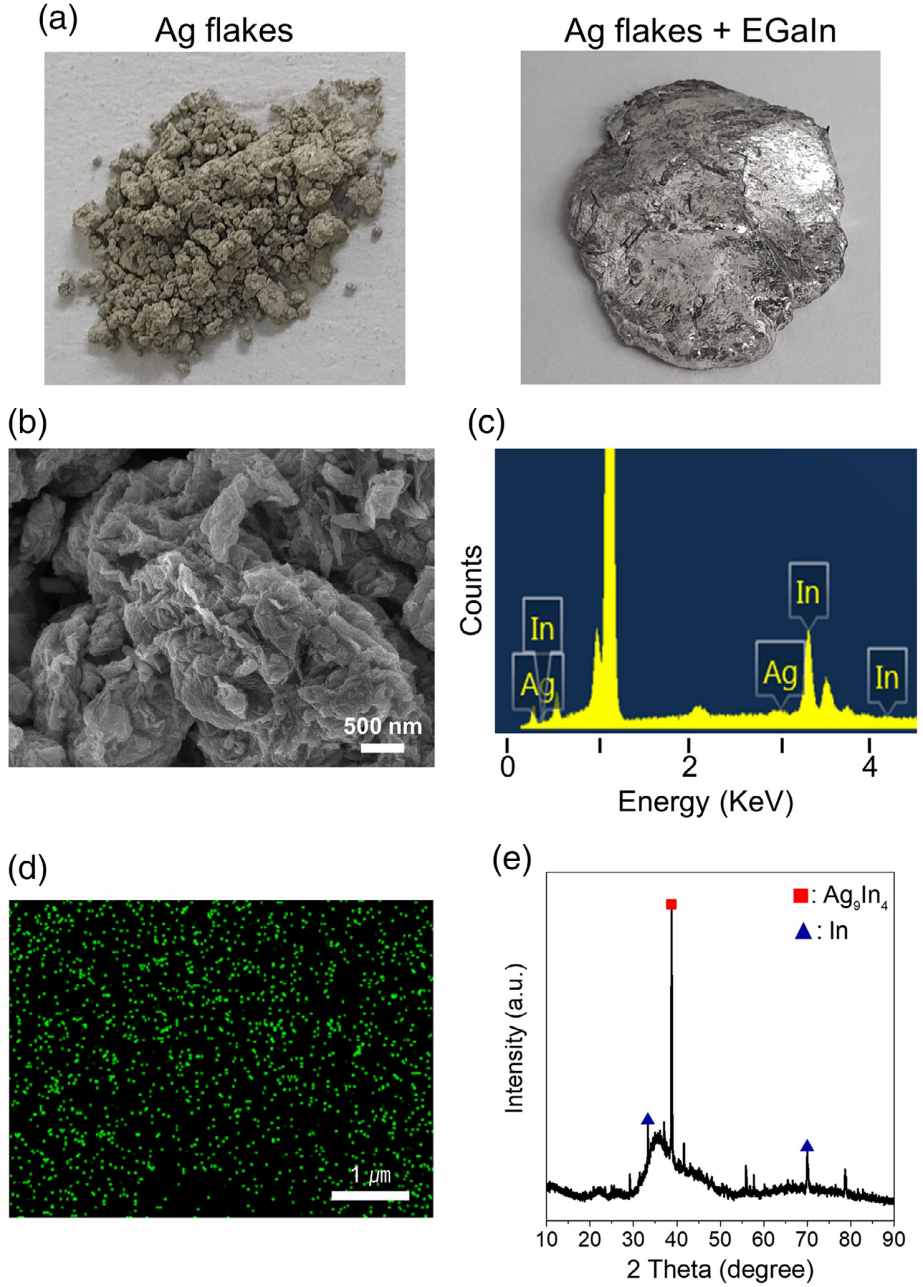
a) Photographs of Ag flakes and a mixture of Ag flakes and EGaIn bulk metals. b) SEM image, c) EDS spectrum, d) EDS Ag mapping image, and e) XRD result for the mixture of Ag flakes and EGaIn bulk metals. The Ag flake–EGaIn mixture was prepared 6 h later after mixing them.

The alloying reactions between the Ag and EGaIn phases are known to take place even at room temperature. In a limited number of previous studies, Ag flakes have been used as a solid metallic additive for fabricating biphasic conductors.^[^ ^18^, ^30^, ^31^ ^]^ The most important prerequisite in forming the biphasic conductor is how to control the degree of alloying reactions consuming the liquid metals. The alloying reactions occurring at room temperature are not adjustable unless the environment is cooled down to extremely low temperatures. Such room‐temperature alloying reactions also proceed further under device operation conditions heated at much over room temperature. As a control experiment, we mixed solid Ag flakes and liquid EGaIn bulk metals. The composition ratio of Ag to EGaIn was set to 9/1 by weight. **Figure** **2**a shows a photograph of the Ag flake–EGaIn mixture 6 h later after mixing them. Interestingly, for the mixture comprising mainly dark‐gray‐colored Ag flakes, the color changed into a shiny light‐gray similar to the color of the EGaIn itself. Distinctively, as seen in the SEM image (Figure 2b), the morphological feature of the disc‐shaped Ag flakes, with a diameter of 1–2 μm, was totally distinct, and rather, highly aggregated particulate clusters were observed in the overall mixture. Figure 2c,d shows the energy‐dispersive X‐ray spectroscopy (EDS)‐based composition and mapping results for the Ag flake–EGaIn mixture. It is clearly observed that the composition of silver diminishes significantly (the atomic ratio of Ag to In was measured as 1/20), even if the silver element is distributed uniformly in the mixture. Taken into consideration that the EDS analysis is limited to the surface of the specimen, it can be deduced that the Ag phase is surrounded by the thick Ga–In phase, and the Ag metals turns into the alloy metal phase. Figure 2e shows the X‐ray diffraction (XRD) result for the mixture. The mixture is composed of predominantly the Ag_9_In_4_ alloy phase and partly other unassigned subphases. It is rationally presumed that the Ag phase with high chemical reactivity for the indium element forms solid Ag–In alloy phases by extracting the indium element from the liquid EGaIn, and the remnant amorphous gallium‐rich metals still have a liquid property due to the low melting point of the gallium element. The mixture consists of both the solid Ag–In phase and liquid (Ga‐rich) In–Ga phase, showing a shiny color by the outer In–Ga phase. However, the progress of the alloying reactions was not controllable, which was only mitigated by a self‐limited diffusion restriction at a given temperature.

**Figure 3:**
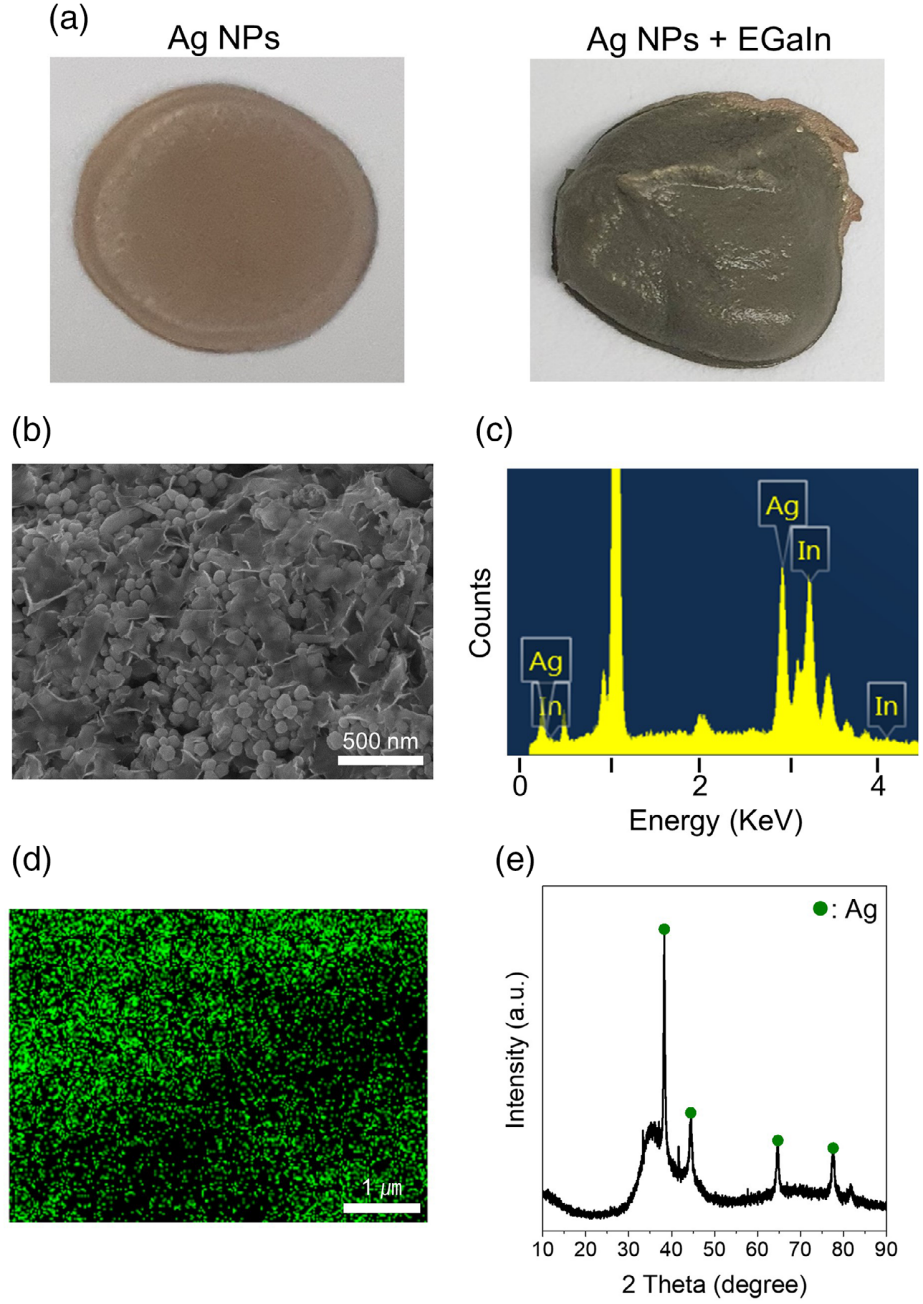
a) Photographs of Ag NPs and a mixture of Ag NPs and EGaIn bulk metals. b) SEM image, c) EDS spectrum, d) EDS Ag mapping image, and e) XRD result for the mixture of Ag NPs and EGaIn bulk metals. The Ag NP–EGaIn mixture was prepared 6 h later after mixing them.

In contrast, for the case of the Ag NPs passivated by long‐chain polymers, the interfacial alloying reactions between the silver and indium elements are circumvented due to the presence of a polymeric passivation layer as a diffusion barrier. As seen in the photographs of the Ag NPs and Ag NP–EGaIn mixture (**Figure** **3**a), the color of the Ag NP–EGaIn mixture is almost identical to the color of the Ag NPs. As shown in the SEM image of the Ag NP–EGaIn mixture, the morphological feature of the Ag NPs is still preserved and a sheath‐like planar morphology, attributable to the liquid EGaIn bulk metals, is observed covering partly the Ag NP assemblies (Figure 3b). This distinctive discrepancy in both mixtures indicates that interfacial alloying reactions between the Ag NPs and EGaIn bulk metals are prevented effectively unlike the case of the Ag flake–EGaIn mixture. This is also supported with the EDS results of the Ag NP–EGaIn mixture (Figure 3c,d). Different from the result of the Ag flake–EGaIn mixture, it appears that much more Ag elements are distributed in the overall mixture, and a higher Ag content was detected with the atomic ratio of Ag to In approaching a value of 11/9. As confirmed in the XRD result (Figure 3e), the peaks by the crystalline Ag NPs and amorphous liquid EGaIn are observed all together without the evolution of any other kinds of alloy phases. Thus, it can be presumed that the interfacial alloying reactions do not proceed in the case of EGaIn/Ag HPs even for a prolonged time, which are triggered only by providing the thermal energy to overcome the activation energy associated with the polymeric diffusion barrier. Thus, the alloying reactions can be controlled depending on the level of thermal energy generated by a kinetically controlled instantaneous laser irradiation reaction. When thermal energy is supplied by a conventional heating method, most liquid EGaIn bulk metals take part in the alloying reactions and the gallium element of liquid EGaIn is fully oxidized, resulting in liquid‐free all‐solid insulating layers.^[^ ^30^ ^]^

**Figure 4:**
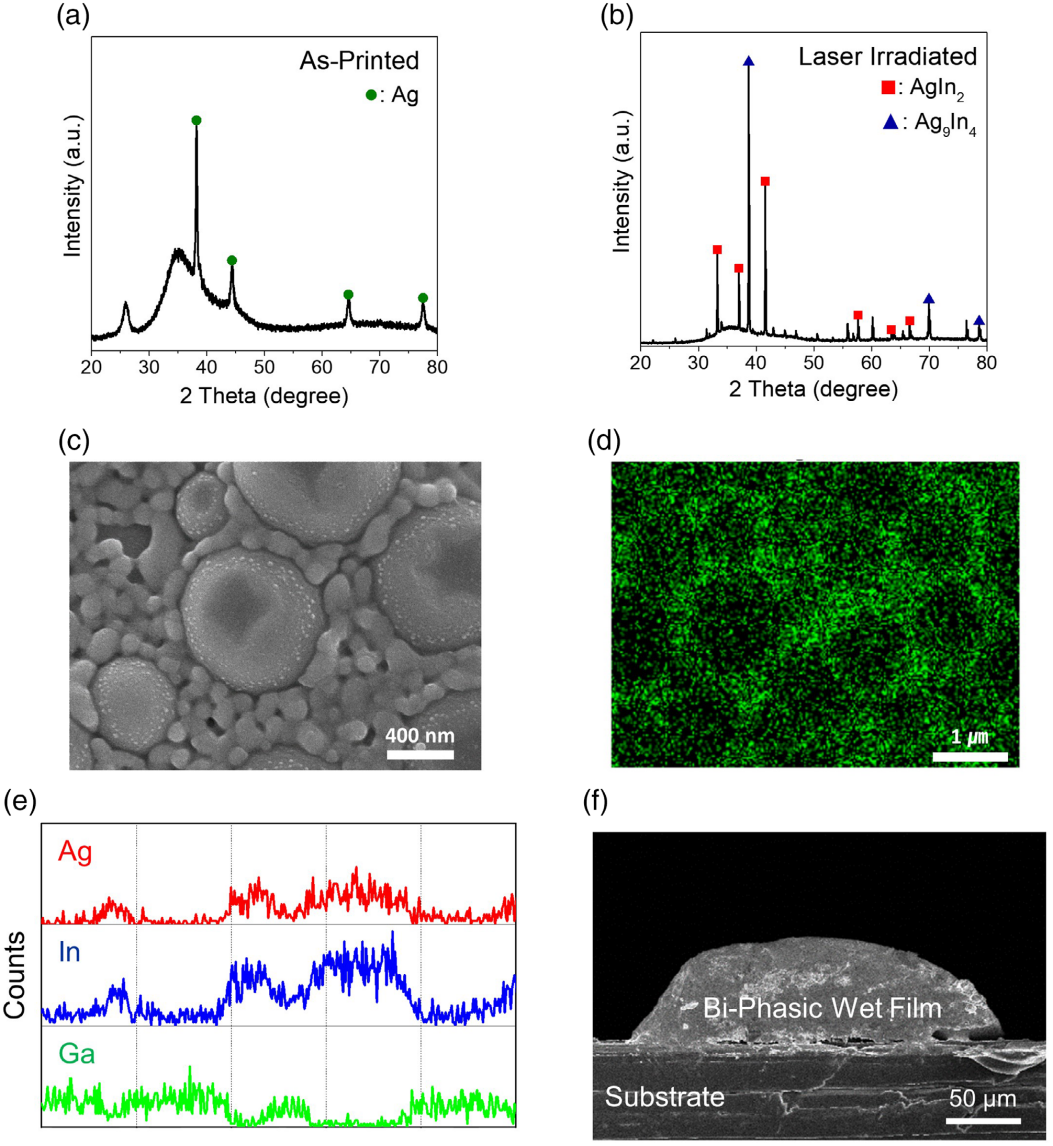
a,b) XRD results for the as‐printed (a) and laser‐irradiated layers (b). c) Top‐view SEM, d) EDS Ag mapping result, and e) EDS Ag, In, and Ga line mapping data for the laser irradiated layers. f) Cross‐sectional SEM image of the laser‐irradiated layer after cutting with a knife. The green laser irradiation process was performed at a scan speed of 200 mm s^−1^ with a laser power of 1.8 W in air.

We formulated inks comprising EGaIn/Ag HPs and printed them using the on‐demand three‐axis programmable dispensing equipment. The as‐printed features were insulated due to the presence of a surface oxide skin layer surrounding the EGaIn MPs.^[^ ^26^, ^27^, ^28^, ^32^ ^]^ Then, the printed features were annealed instantaneously by a green laser irradiation process at a scan speed of 200 mm s^−1^ with a power of 1.8 W. A conductivity reaching a value of 1067 S cm^−1^ was obtained after the green‐laser irradiation process. When the green laser irradiation process was carried out at a scan speed of 400 mm s^−1^, the values in conductivity were measured to be 625.2 and 760.4 S cm^−1^ at laser power conditions of 2.5 and 3.4 W, respectively. The optical energy dose increases accordingly by decreasing the scan speed or increasing the laser power. The conductivity increased correspondingly with the generation of more thermal energy under a higher optical energy dose. This implies that the rupture of the surface oxide layer is thermally driven, along with the thermal activation of the interfacial alloying reactions and the thermal decomposition of the polymeric diffusion barrier. As confirmed in the XRD results for the as‐printed and laser‐irradiated layers (**Figure** **4**a,b), after the laser irradiation process (at a scan speed of 200 mm s^−1^ and a laser power of 1.8 W), an evident evolution was observed of the peaks attributable to the Ag_9_In_4_ and AgIn_2_ alloy phases, while the broad peaks by the liquid metals were still observed in the range of 25–50°.

Figure 4c shows the top‐view SEM image of the laser‐irradiated layer. The low‐magnification top‐view SEM images of as‐printed and laser‐irradiated layers are shown in Figure S4, Supporting Information. It is observed that upon a laser irradiation process, the micrometer‐sized domains are partly dented and interconnected by 75 nm‐thick particulate outer layers. As for the EDS‐based Ag element mapping image, the Ag content is highly localized around the micrometer‐sized domains, and the subtle Ag element is observed inside the micrometer‐sized domains (Figure 4d). The EDS elemental profile data for the Ag, In, and Ga elements suggest that the internal micrometer‐sized domains are composed mainly of In‐deficient liquid metals, and the interconnected particulate layers surrounding the In‐deficient liquid metals comprise solid Ag–In alloys. This implies that the In element diffuses outward from the EGaIn MPs to form the outer Ag–In alloy particulate layers, and the counter inner diffusion of the Ag element occurs slightly toward the In‐deficient In–Ga liquid metals. Meanwhile, the surface oxide skin layer was thermally ruptured, and the polymeric diffusion barrier of the Ag NPs was thermally decomposed. The solidification by the formation of the surficial Ag–In alloy phases would increase the density of the micrometer‐sized domains, which results in a volumetric contraction represented by a dented morphology shown in Figure 4c. This characteristic spatial distribution of the solid and liquid phases gives rise to the generation of a biphasic microstructure in which individual liquid‐phase In–Ga alloys are surrounded by solid‐phase Ag–In alloys. Interestingly, even after cutting the green‐laser annealed layer with a knife, the feature of the as‐printed layer was maintained even with the formation of the bulk wet film. The continuous bulk wet film was created by a flood of internal In–Ga liquid metals out of the micrometer‐sized domains, which is notably still captured by the internetworked solid alloy phases, maintaining its overall morphology (Figure 4f and S5, Supporting Information).

### 2.3 Thermal Profile in Laser‐Processed Biphasic Conductor

A significant advantage of using an instant laser irradiation process is providing thermal energy in a timescale of below 1–10 s (for the case of a single scan). All thermal events, including heat generation, heat consumption by endothermic reactions, and heat loss toward outer surroundings, occur simultaneously in an extremely short timeframe. Notably, such thermal events take place in all localized spots around the Ag NPs so that an increment in temperature in the specimen is not high enough to result in thermal damage to the underlying polymeric substrates. Figure S6a, Supporting Information, shows a real‐time temperature profile measured using a high‐resolution infrared camera at a framerate of 50 Hz, while irradiation with the green laser was performed consecutively 100 times with line spacing of 23 μm. The maximum temperature only reaches a value of ≈140 °C, which is much lower than the glass transition temperature of the 3D‐printed polymeric substrate used in this study. This implies that even though thermal energy is accumulated by the consecutive green laser irradiation process, the temperature at the surface of the laser‐annealed biphasic conductors does not increase over 150 °C by an ultrafast heating/cooling behavior. The gradient‐free temperature uniformity is also confirmed in the real‐time image and video (Figure S6b and Movie S1, Supporting Information).

**Figure 5:**
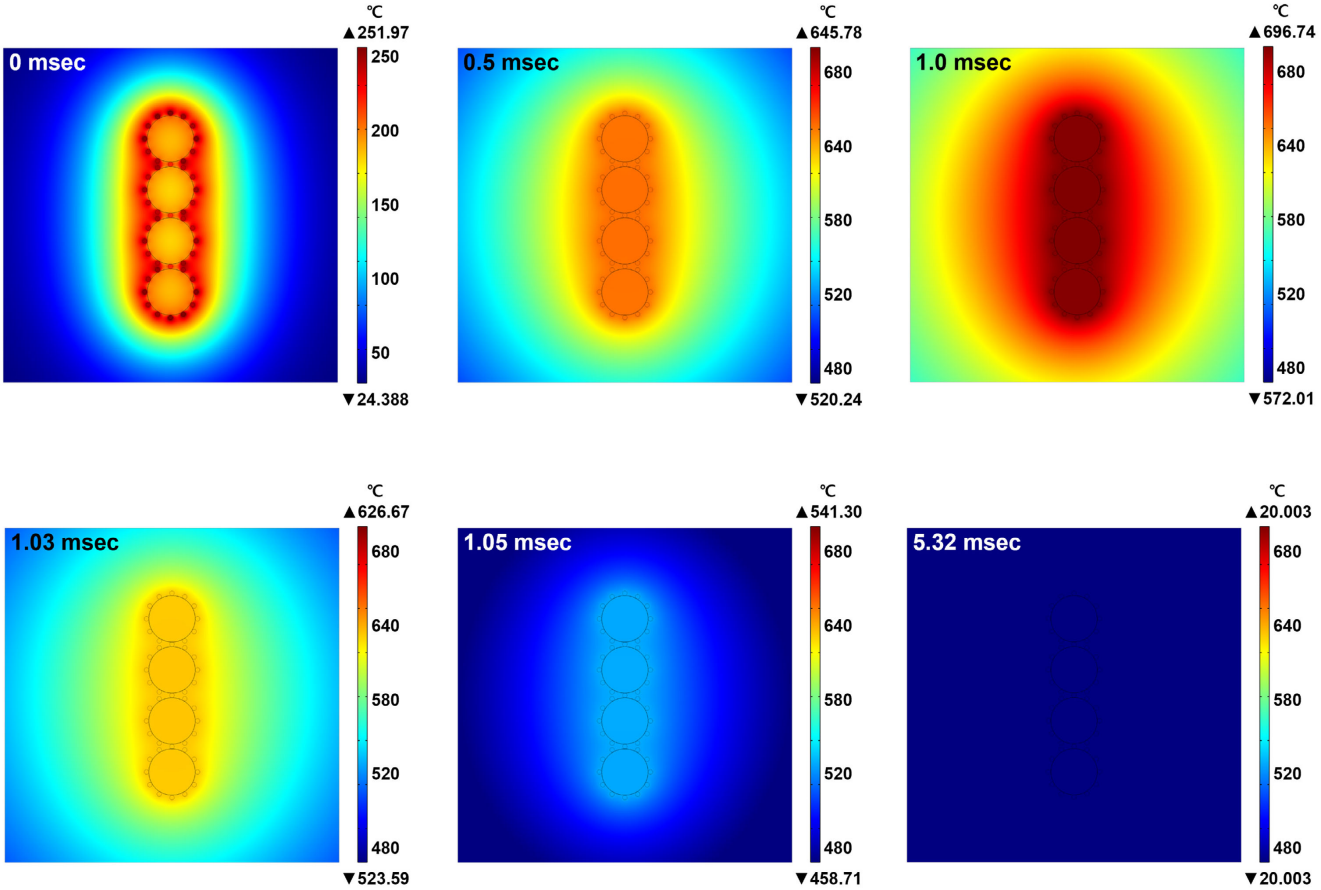
Transient temperature distributions in the EGaIn/Ag HPs upon green laser irradiation.

To clarify the instant heating/cooling behaviors in the EGaIn/Ag HPs by the green laser irradiation, the transient thermal response of the EGaIn/Ag HPs was calculated with a time‐dependent heat conduction equation subjected to thermal energy flux from surface heat sources. To simplify the simulation model, four EGaIn MPs with a diameter of 1 μm were decorated with 100 nm‐sized Ag NPs, as shown in **Figure** **5**. Upon laser processing at an optical power of 1.8 W and a scan speed of 200 mm s^−1^, Ag NPs with higher absorption coefficient (relative to the EGaIn MPs) mainly absorb the laser irradiation energy to initiate significant heat generation within the diffraction limit around NPs. This strong energy confinement phenomenon is attributed to resonant coupling between the free electrons in the Ag NPs and the electromagnetic wave with a specific wavelength of 532 nm, which causes an efficient plasmonic absorption (collective motion of electrons) of photon energy to induce thermal dissipation in an extremely confined space. It should be noted that the metal–dielectric–metal junctions formed at the closely located Ag NPs can further enhance the plasmonic heat generation efficiency by causing a dissipative interaction (attraction and repulsion) between the light‐induced oscillating surface dipoles of the NPs, leading to dramatic thermal effects that cannot be achieved in bulk materials.^[^ ^33^, ^34^ ^]^ The generated intensive photothermal energy was immediately transferred to the EGaIn MPs with high thermal conductivity and increased the temperature up to ≈700 °C within 1 ms. The huge temperature gradient between the EGaIn/Ag HPs and air surroundings enables drastic quenching of instantly generated heat, enabling the EGaIn/Ag HPs to cool down to room temperature in a timescale of less than 10 ms.^[^ ^35^, ^36^, ^37^ ^]^

This instant local heating/cooling behaviors prevent undesirable oxidation reactions forming insulating oxide phases. It has been reported that thermal oxidation reactions of EGaIn liquid metals occur predominantly for the Ga element.^[^ ^32^ ^]^ Figure S7a,b, Supporting Information, shows the X‐ray photoelectron spectroscopy (XPS) Ga 3d spectra for the as‐printed and green‐laser‐irradiated layers. For the as‐printed layer, both major peaks attributable to Ga^3+^ in Ga_2_O_3_ and Ga^0^ in Ga metal were detected at binding energies of 19.6 and 17.3 eV, respectively.^[^ ^38^ ^]^ According to a semiquantitative analysis, the atomic ratio of Ga to Ga_2_O_3_ was measured to be 30/70. Considering the measurement depth in the XPS analysis, it is presumed that a Ga_2_O_3_ layer a few nanometers thick was formed as a native oxide layer. After the green laser irradiation process, oxidation reactions proceed further. The atomic ratio of Ga to Ga_2_O_3_ was measured to be 7/93. It is believed that the thickness of the surface oxide skin layer is still limited to a value on a scale of a few nanometers. The XPS Ag 2p spectrum for the green laser‐annealed layer is shown in Figure S7c, Supporting Information. The spectrum is deconvoluted into three peaks by the Ag^0^ in the Ag metal, Ag^+^ in Ag_2_O, and Ag^2+^ in AgO positioned at binding energies of 367, 366.3, and 366.8 eV, respectively.^[^ ^39^ ^]^ The atomic fraction of the pure metal phase is calculated to be 76.2 at%, indicative of critical suppression in oxidation reactions of the Ag metal phase. These results suggest that inappropriate oxidation reactions are mitigated in the laser‐assisted thermal alloying reactions, making it possible to facilitate highly functioning solid/liquid biphasic conductors with high purity in metal content. It can be presumed that the slightly oxidized bulk structure is generated with a formation of air‐stable alloy phases. The micrometer‐sized EGaIn droplets are oxidized significantly in air, but, the oxidation reaction in bulk EGaIn liquid metal is self‐limited so its chemical/physical properties are preserved even for a prolonged time in ambient. In order to investigate the stability in air of laser‐annealed biphasic conductors, we performed XRD analysis for the sample stored in air for 2 months (Figure S8, Supporting Information). It was revealed that a significant change in crystalline phase is not observed with an evolution of silver indium alloys phases identical to those of the as‐fabricated sample.

### 2.4 Synergetic Advantage of Solid/Liquid Phases in Biphasic Conductor

**Figure 6:**
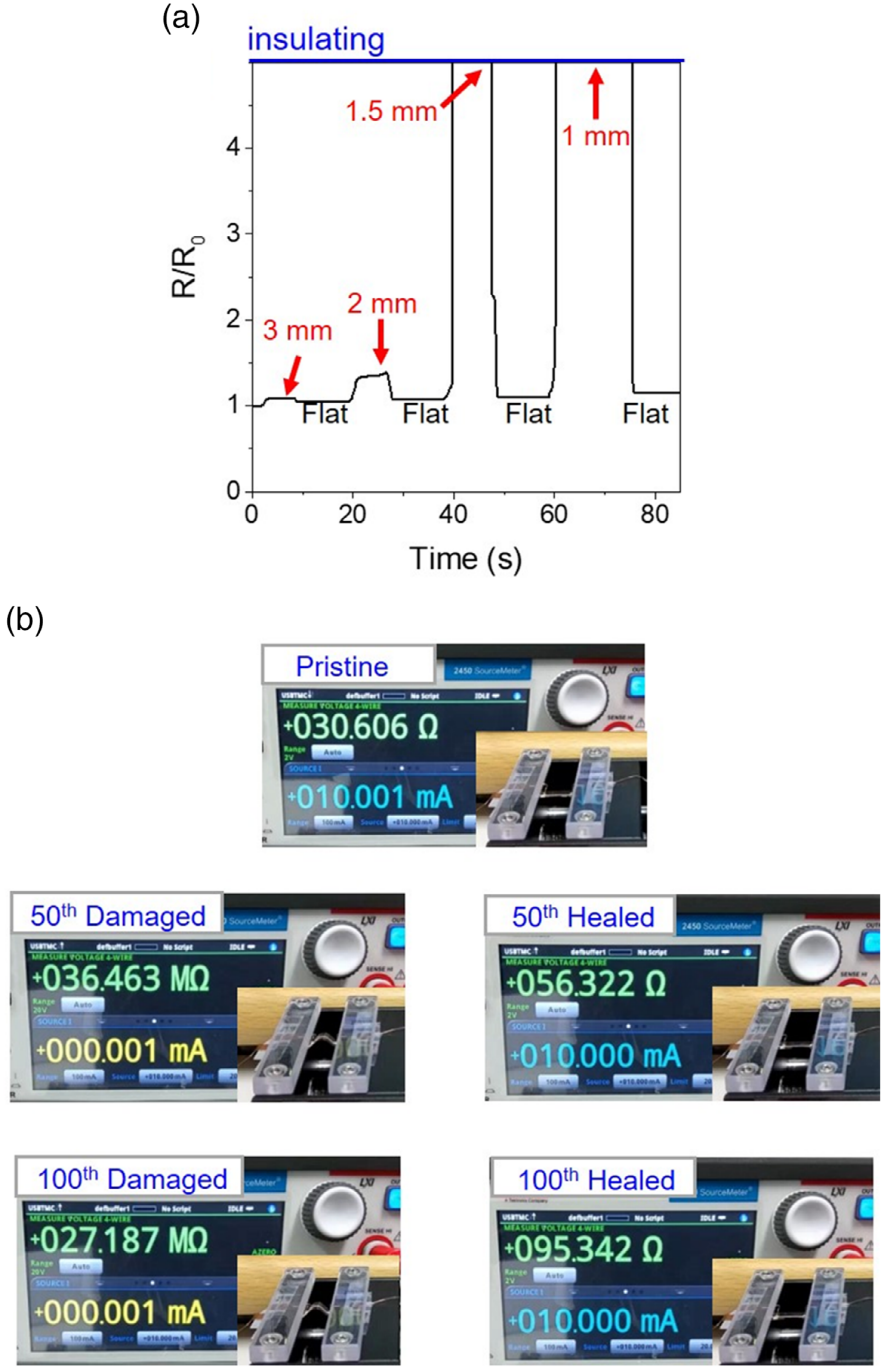
a) Variation in resistance depending on the bending radius for the biphasic conductor fabricated on the polyimide substrate; b) photographs showing the resistance for the pristine, 50th‐cycled and 100th‐cycled biphasic conductor. The green laser irradiation process was performed at a scan speed of 200 mm s^−1^ with a laser power of 1.8 W in air.

An advantageous characteristic of the liquid phase is that they can be merged instantly when being in contact even physically. This implies that in the case in which individual biphasic metallic layers are in contact, their cross‐sectional interface can be merged into a single material. In other words, the broken conductor lines can be healed without an involvement of additional stimuli, such as temperature and pressure. The polymers with polar groups (enabling a strong secondary bonding) can be healed with a simultaneous provision of temperature and pressure,^[^ ^40^, ^41^, ^42^ ^]^ but, the solid metal and ceramic are not ever healed because of a lack of healable functional chemical groups. We carried out the outer bending test for the biphasic metallic layer formed on the flat polyimide substrate. A tensile strain is applied during the outer bending test, the level of which is proportional to the thickness of the layer formed on top of the given substrate. Thus, the flexible layer should be thin enough to reduce the level of tensile strain under outer bending conditions. However, as the 3D‐printable ink formulated in this study has a highly viscoelastic rheological property, the biphasic metallic layer has a thickness as thick as 76.6 μm. This leads to a strain of 5.05% at a bending radius of 1.5 mm (the thickness of the polyimide substrate used in this study is 75 um).^[^ ^43^ ^]^ The solid metal compartment in biphasic conductors do not accommodate such a high level of tensile strain. As seen in the variation of the resistance as a function of the bending radius, the resistance increased slightly at a bending radius of 3 and 2 mm and the electrical properties were not measured at a bending radius below 1.5 mm (**Figure** **6**a). Notably, it is clearly observed that the resistance was almost completely recovered when the biphasic conductor became subsequently flat after being bent at a bending radius below 1.5 mm. The holding time for being unbent was 5 s. This implies that the broken conductive layer is healed in a timescale of a few seconds. Figure 6b, S9, and Movie S2, Supporting Information, show the 100 times cycling test result at a bending radius of 1.5 mm. It appears that the resistance recovered continuously, even though it increases gradually as a function of the number of cycling. This healing capability is also confirmed even in the case in which the biphasic metallic layer is cut on purpose and subsequently comes into physical contact (Figure S10 and Movie S3, Supporting Information). The resistance was recovered repeatedly to the pristine value.

**Figure 7:**
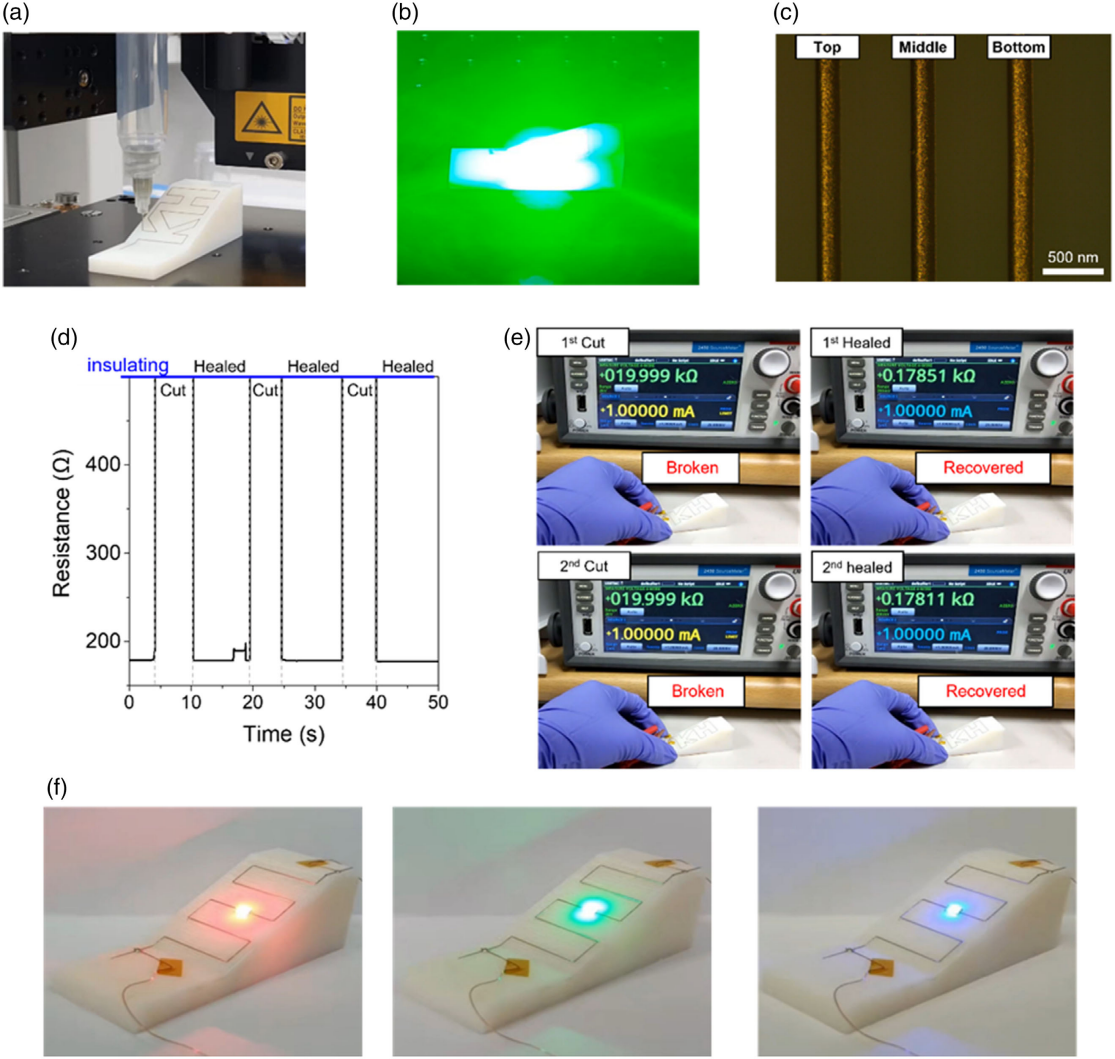
Photographs showing the a) 3D printing process and b) surface conformal laser irradiation process. c) Optical microscopy image of the 3D biphasic metallic feature fabricated on top of the 3D polymeric structure. d) Variation in the resistance measured while cutting and contacting repeatedly the 3D biphasic conductor. e) Photographs showing the resistance recovered to the pristine value after being healed. f) Photographs showing the chip bonding characteristic of the 3D biphasic conductor. The green laser irradiation process was performed at a scan speed of 200 mm s^−1^ with a laser power of 1.8 W in air.

**Figure** **7**a,b and Movie S4, S5, Supporting Information, show the photographs and videos of 3D printing and surface‐conformal green laser irradiation processes on top of the 3D polymeric structure with a slope of 26.6° and a height of 20 mm. As seen in Figure 7c, the 3D biphasic metallic features were created along the preformed 3D structure, and their linewidths were almost identical at all positions (the deviation in the linewidth was only 7.4 μm for all vertical positions). As seen in Figure 7d,e and Movie S6, Supporting Information, the 3D biphasic metallic features were healed instantly after being cut by the knife, indicative of the coexistence of the solid and liquid metallic phases. In the conventional printed circuit board (PCB) electronics, a solder reflow process at elevated temperatures is indispensably required to build intact electrical contacts between loaded chips and conductive lines. Notably, the biphasic conductor has a semiliquid property by itself so that an additional soldering process at a high temperature is not necessary. The interconnection of chips is achievable simply by placing the chips on top of the biphasic conductive features. As seen in Figure 7f and Movie S7, Supporting Information, light‐emitting diode (LED) chips with different operational voltages were chip bonded easily by loading them on top of the 3D biphasic conductive lines. The gentle pressure of placing the LED chips gives rise to an overall flood of the liquid phase (Figure S11, Supporting Information), which enables for a conformal electrical contact with rigid chip components while maintaining 3D structural geometry.

## 3 Conclusion

We have synthesized multidimensional EGain/Ag HPs by decorating the surface oxide‐capped EGaIn particles with size‐tuned Ag nanoparticles. The Ag nanoparticles have the crucial role of absorbing green wavelength photons. It was elucidated that the thermal energy converted from the optical energy triggers chemical/physical evolution at the interface between the Ag nanoparticles and EGaIn particles. Upon the green laser irradiation process, the gallium‐rich liquid metallic cores were surrounded by the solid alloy compartments, generating the 3D biphasic conductors. According to the photothermal simulation and in situ infrared camera observation results, the alloying reactions were completed instantly with heat dissipation toward the outer surroundings. This enables for the suppression of undesirable side reactions (oxidation reactions), along with the activation of desirable target reactions (alloying reactions). It was demonstrated that biphasic conductors can be fabricated by successive 3D printing and 3D surface conformal green laser irradiation processes, exhibiting electrical properties that has not been achievable in conventional solid metallic conductors.

## 4 Experimental Section

4.1

4.1.1

##### Raw Materials

Poly(vinylpyrrolidone) (PVP, M.W. 10 000 g mol^−1^, Aldrich), ethylene glycol (EG, >99.5%, Junsei), silver nitrate (99.5%, Junsei), eutectic gallium indium alloy (EGaIn, Indium Corporation), terpineol (60–85%, Aldrich), and ethanol (Duksan) were purchased and used as received without additional purification. The compositional ratio of EGaIn was 78.6 wt% Ga and 21.4 wt% In.

##### Synthesis of Hierarchical Multidimensional Particles

To synthesize the Ag nanoparticles, we added the PVP and silver nitrate to 150 mL of EG and heated at 120 °C for 1 h. The compositions of the reaction solutions were regulated to synthesize the Ag nanoparticles with a high optical absorption property at a wavelength of 532 nm. The reaction solution was cooled down to room temperature, and the particles were collected by centrifugation. The resulting Ag nanoparticles were dispersed in ethyl alcohol and stored in air. Next, 1.2 g of EGaIn bulk metal was mixed with 10 g of ethyl alcohol, and the liquid metal particles were synthesized by the tip sonication process. The amplitude of tip sonication process was set to be 60%, and the sonication time was 2 min. By increasing the sonication time up to 2 min, the average size of liquid metal droplets became smaller and the size distribution of them became narrower, which determined the morphological features of surface‐oxidized EGaIn particles (Figure S12, Supporting Information). The EGaIn particles were collected by centrifugation. The EGaIn particles were dispersed in ethyl alcohol with the addition of additional PVP and mixed with the synthesized Ag nanoparticles. The multidimensional hierarchical EGaIn/Ag particles were collected by centrifugation, and the supernatant including the free Ag nanoparticles was eliminated. All synthesis procedures were carried out in air.

##### 3D Printing and Surface Conformal Green Laser Irradiation Processes

The hierarchical metallic inks were formulated by dispersing the multidimensional EGaIn/Ag particles with a solid loading of 92 wt%. The solid loading was regulated to have a high viscoelasticity for 3D direct ink writing on flat polyimide (PI, thickness (*t*) = 75 μm, Kapton film 300HN, Teijin DuPont Films) substrates or 3D polymeric structures which were made by a polyjet 3D printer (Objet30 Pro, Stratasys). The highly viscous inks were printed using a programmable dispenser (Image Master 350PC Smart, Musashi) with a nozzle with an inner diameter of 200 μm. After drying the printed features, the 3D surface conformal green laser irradiation process was carried out using the 3D laser scanner for which a laser source and *f*–*θ* lens were equipped with a wavelength of 532 nm and a focal length of 275.2 mm, respectively. The spot size of the laser beam was 230 μm in diameter. The 3D laser scanning process was performed by modulating optically the trajectory of the irradiated photons, keeping a laser focus along the surface of the 3D structures. The scanned lines were overlapped with an interval of 23 um. The laser power and scan speed were regulated with values of 1.8–3.4 W and 200–400 mm s^−1^, respectively. All laser scanning processes were performed in air.

##### Optical Simulation

Finite‐difference time‐domain (FDTD) simulations (FDTD Solutions, Lumerical) on Ag nanoparticles were performed to explore their scattering properties. The optical constants of the Ag material were derived from the previous literature.^[^ ^44^ ^]^ Broadband (200–1000 nm in 5 nm steps) normally incident plane waves and a frequency‐domain power monitor were used to acquire the forward and backward scattered fields, respectively. Electric and magnetic scattered fields were obtained via a total‐field scattered‐field method. Then, the obtained absorption cross section was divided by the diameter of the Ag nanoparticles to determine the absorption efficiency. The spatial resolution was set to 1 nm in all directions.

##### Thermal Simulation

Simulation for the transient temperature distribution of EGaIn/Ag particles under laser irradiation was carried out with the finite‐element method based on commercial software (COMSOL Multiphysics – heat transfer module). The governing equation for the time‐dependent heat transfer process can be defined as follows.

(1) $$Q=\rho C\frac{\partial T}{\partial t}+\rho C\cdot \nabla T-\nabla \cdot \left(k\nabla T\right)$$

where *Q* is the surface heat source term; *ρ* is the material density; *k* is the thermal conductivity; *c* is the specific heat capacity; *T* is the temperature. To calculate the term of the surface heat source, we assumed that the Ag nanoparticles absorb most of the irradiated green laser energy considering the optical properties such as the absorption coefficient and transmittance at a wavelength of 532 nm. The simulation geometry was simplified by modeling four EGaIn particles with a diameter of 1 μm, which were surrounded respectively in a symmetric configuration by 12 Ag nanoparticles with a diameter of 100 nm. The empty space between the EGaIn particles and Ag nanoparticles was assumed as air medium. The representative properties of the materials applied in this simulation are described in Table S2, Supporting Information.

##### Characterization

The conductivity of the printed and laser‐irradiated biphasic conductors was measured by a digital sourcemeter (2450 SourceMeter, Keithly) with a four‐probe measurement setup. The both ends of printed and laser irradiated biphasic conductors were attached with Cu wires (diameter: 0.2 mm, Nilaco Corporation) and silver epoxy. The dimensions of printed and laser irradiated biphasic conductors were observed by optical microscopy (BX53M, Olympus) and SEM (MERLIN, Carl Zeiss). The crystal structural and chemical information were obtained by XRD (D8 Advance, Bruker) and XPS (XPS, K‐Alpha, Thermo Fisher Scientific) analyses for cast samples, respectively. The laser processing condition for cast samples was optimized again based on the conductivity data. The temperature profiles during the green laser irradiation process were monitored using a high‐resolution infrared camera (A655SC, FLIR) at a framerate of 50 Hz. A Fourier transform infrared spectrometer (INVENIO R, Bruker) equipped with a gold diffuser‐coated integrating sphere (A562‐G/Q, Thorlabs) and mercury telluride detector was used to obtain the absorptivity/emissivity spectrum at thermal radiation wavelengths of 5–15 μm. The obtained “apparent” temperature of each sample was calibrated using its measured emissivity to determine the actual temperature.

## Conflict of Interest

The authors declare no conflict of interest.

## Supporting information

Supplementary Material

## Data Availability

The data that support the findings of this study are available from the corresponding author upon reasonable request.
