# Supplementary material for: A Surface Conformal Laser‐Assisted Alloying Reaction for 3D‐Printable Solid/Liquid Biphasic Conductors
Source: Small Sci. 2023 Feb 3;3(3):2200089. doi: 10.1002/smsc.202200089 (PMC11935982; doi:10.1002/smsc.202200089)
Supplement: Supplementary file 1 — Supplementary Material [file SMSC-3-2200089-s001.zip › smsc202200089-sup-0002-SuppData-S2.pdf]

Supporting Information

A Surface Conformal Laser-Assisted Alloying Reaction for 3D-Printable Solid/Liquid Biphasic Conductors

Jiyun Shim, Yeon Uk Kim, Young-Bin Kim, Seul Gi Ji, Yeon Ju Kim, Yejin Jo, Eun Jung Lee, Do-Gyeong Yuk, Su Yeon Lee, Sun Sook Lee, Sun-Kyung Kim, Hyung-Seok Kim, Jung Hwan Park, Sunho Jeong**

J. Shim, Y. J. Kim, Dr. Y. Jo, Prof. S. Jeong
Department of Advanced Materials Engineering for Information and Electronics, Integrated Education Institute for Frontier Science & Technology (BK21 Four), Kyung Hee University, Yongin-si 17104, Republic of Korea
E-mail: sjeong@khu.ac.kr

Dr. H.-S. Kim, Prof. S. Jeong
KHU-KIST Department of Converging Science and Technology, Kyung Hee University, Seoul, 02447, Republic of Korea

Y. U. Kim
Department of Mechanical Design Engineering, Kumoh National Institute of Technology, 61 Daehak-ro, Gumi, Gyeongbuk 39177, Republic of Korea

E. J. Lee, Dr. S. Y. Lee, S. G. Ji, Dr. S. S. Lee
Division of Advanced Materials, Korea Research Institute of Chemical Technology, Daejeon 34114, Republic of Korea

Y.-B. Kim. Prof. S.-K. Kim
Department of Applied Physics, Kyung Hee University, Yongin-si 17104, Republic of Korea

D.-G. Yuk, J. H. Park
Department of Aeronautics, Mechanical and Electronic Convergence Engineering, Kumoh National Institute of Technology, 61 Daehak-ro, Gumi, Gyeongbuk 39177, Republic of Korea
E-mail: parkjh1151@kumoh.ac.kr

Table S1. Details on the reaction solutions for synthesizing the Ag nanoparticles

		Silver Nitrate	PVP	Ethylene Glycol
Ag-#1 NPs	Weight (g)	6.4	40.5	135.1
Ag-#2 NPs		6.4	15.2	135.1
Ag-#3 NPs		6.4	10.1	135.1
Ag-#1 NPs	Concentration (M)	0.25	0.03	-
Ag-#2 NPs		0.25	0.01	-
Ag-#3 NPs		0.25	0.007	-

Table S2. Representative material properties used for the transient photothermal simulation

Density (kg/m^3):<br>Ag NP / EGaIn MP / Ga_2O_3	10,490 / 6,280 / 6,440
Specific heat capacity(J/(kg·k)):<br>Ag NP / EGaIn MP / Ga_2O_3	235 / 404 / 560
Thermal conductivity (W/(m·k)):<br>Ag NP / EGaIn MP / Ga_2O_3	416 / 26.6 / 11

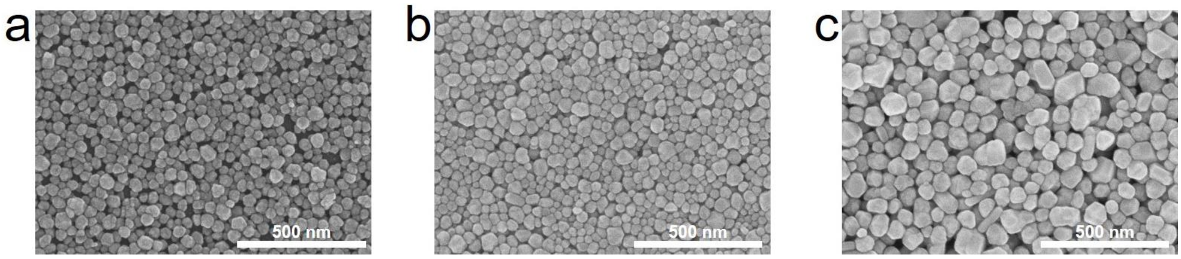

Figure S1. SEM images of the Ag-#1, Ag-#2 and Ag-#3 NPs.

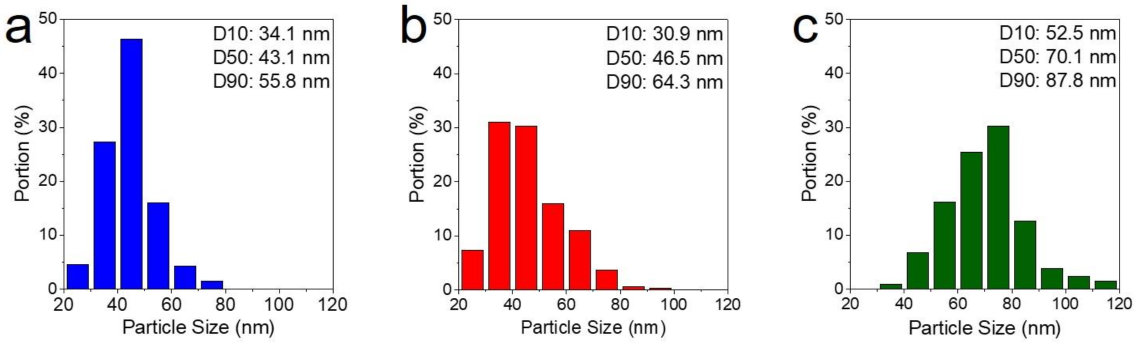

Figure S2. Histograms of the diameters for the Ag-#1, Ag-#2 and Ag-#3 NPs.

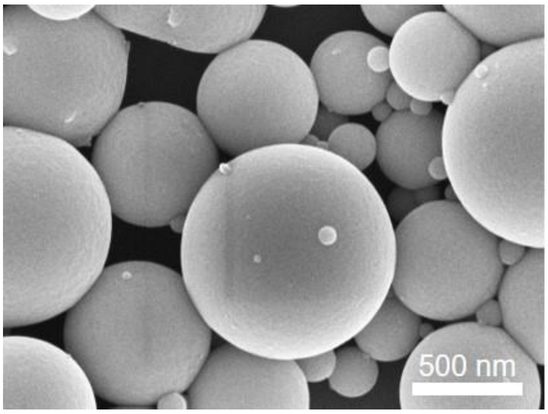

Figure S3. SEM image of the EGaIn MPs.

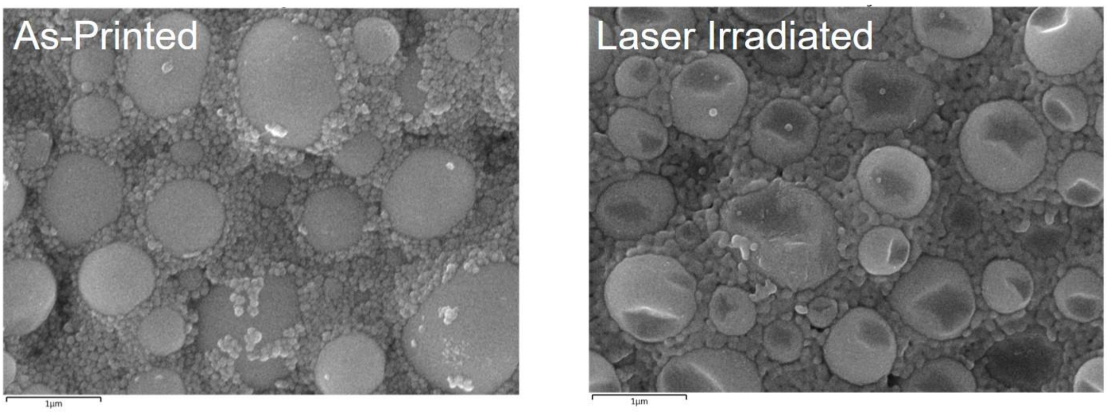

Figure S4. Low magnification top-view SEM images for as-printed and laser irradiated layers. The Ag nanoparticles positioned on top of the printed layer swept away, by a shear force applied along the inner surface of the nozzle during a nozzle printing process.

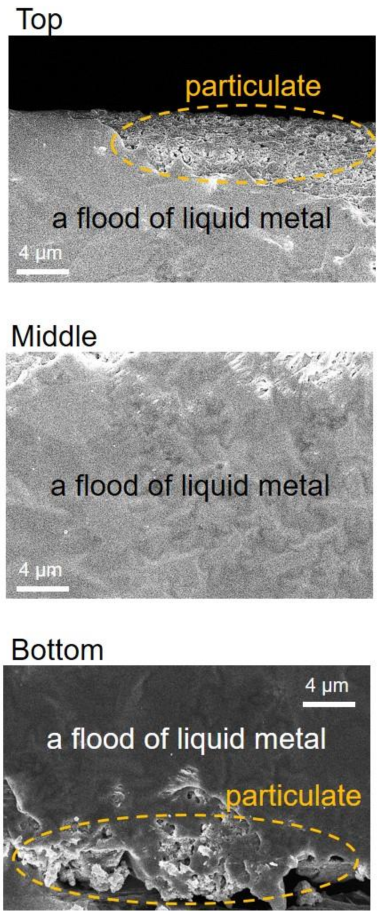

Figure S5. Cross-sectional SEM images of the laser irradiated layer after cutting with a knife.

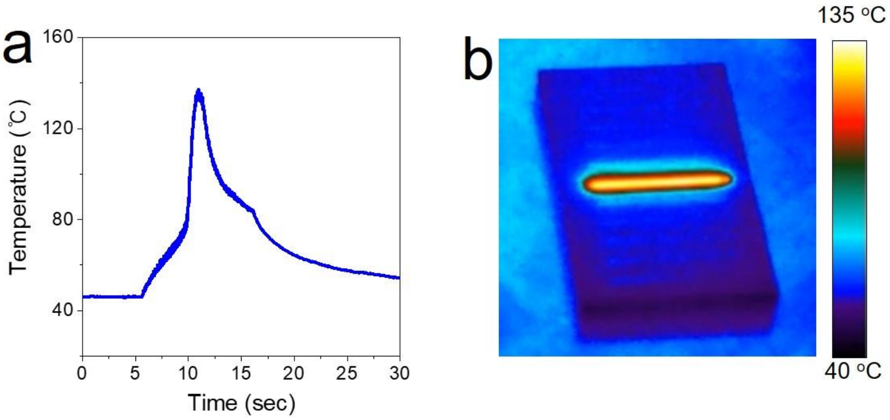

Figure S6. (a) Temperature profile and (b) real-time thermal image measured during the green laser irradiation process.

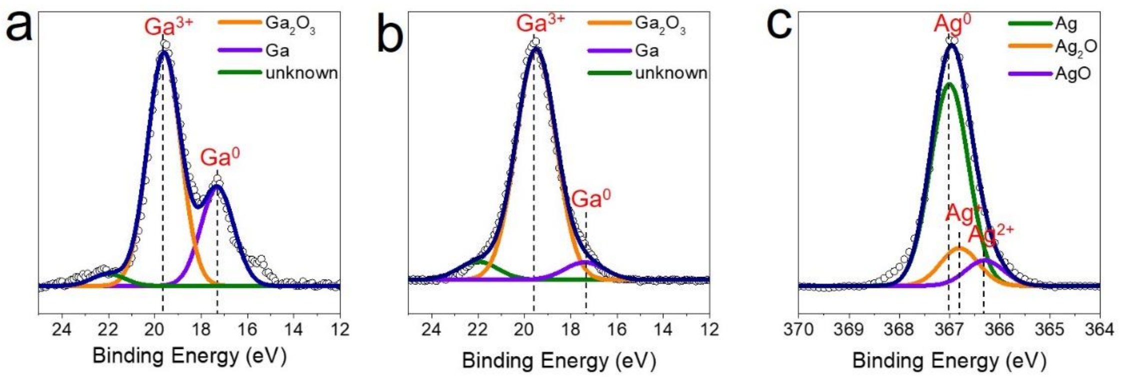

Figure S7. XPS Ga 3d spectra for the (a) as-printed and (b) laser irradiated layers; (c) XPS Ag 2p spectrum for the laser irradiated layer.

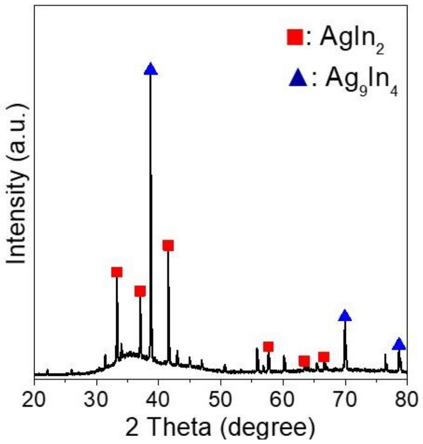

Figure S8. XRD result for the bi-phasic conductor stored in air for 2 months.

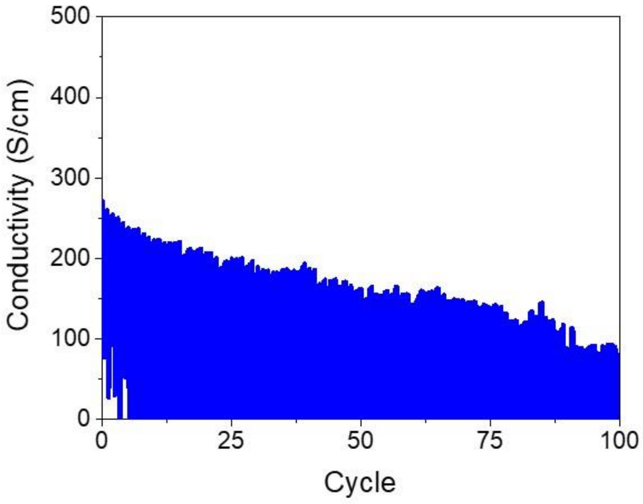

Figure S9. Variation in resistance during a repeated cycling test at a bending radius of 1.5 mm for the bi-phasic conductor fabricated on the polyimide substrate. A slightly lower conductivity was measured for the bi-phasic conductor fabricated on top of the flat polyimide substrate, because of a photothermal damage of the semitransparent polyimide itself. The green laser irradiation process was performed at a scan speed of 200 mm/sec with a laser power of 1.8 W. The bi-phasic conductor is healed by a contribution of liquid compartment without a critical leakage out of the patterned geometry. While a repeated bending test is conducted, the new outer surface of liquid compartment is indispensably created, which undergoes subsequent oxidation reactions. This results in a gradual increment in resistance during a repeated bending test.

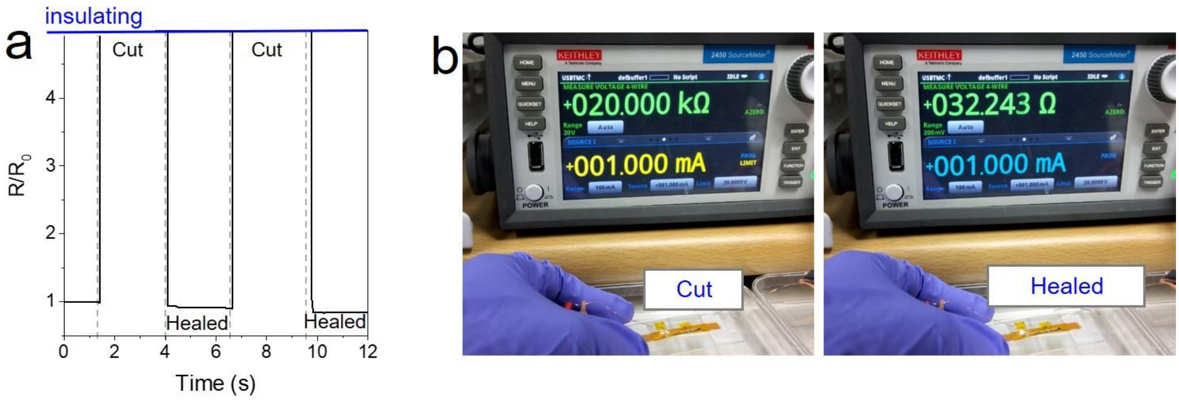

Figure S10. (a) Variation in the resistance measured while cutting and contacting repeatedly the bi-phasic conductor; (b) Photographs showing the resistance recovered to the pristine value after being healed.

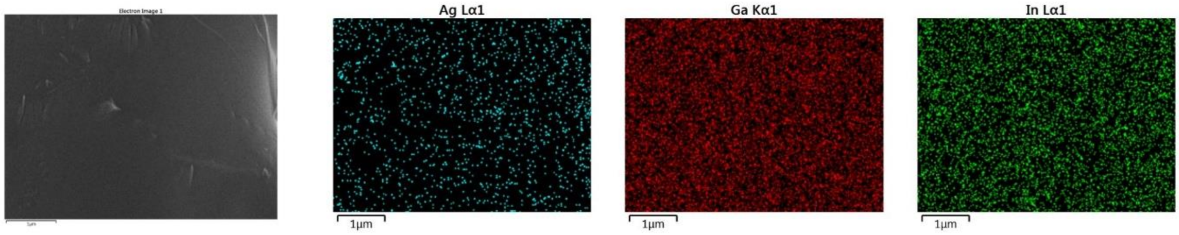

Figure S11. SEM image and EDS elemental mapping results for the 3D bi-phasic conductive feature where the LED chip was loaded.

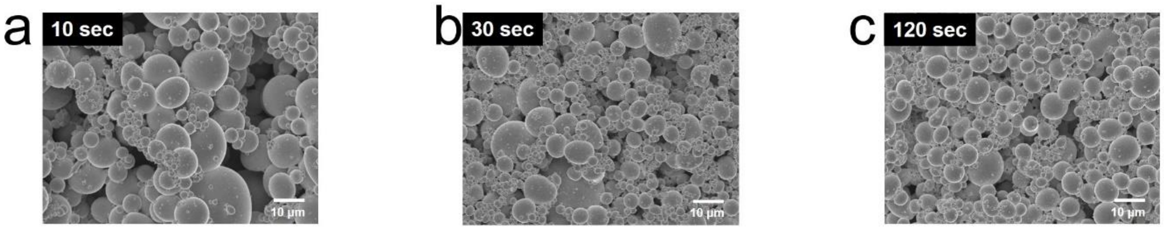

Figure S12. SEM images of EGaIn particles synthesized in this study by a tip sonication during a different sonication time.

Movie S1. A video of showing the real-time measurement of the heating temperature during a green laser irradiation process.

Movie S2. A video of showing the resistance recovered to the pristine value after being bent and healed (flat bi-phasic conductor).

Movie S3. A video of showing the resistance recovered to the pristine value after being cut and healed (flat bi-phasic conductor).

Movie S4. A video of showing the 3D printing process.

Movie S5. A video of showing the surface conformal green-laser irradiation process.

Movie S6. A video of showing the resistance recovered to the pristine value after being healed (3D bi-phasic conductor).

Movie S7. A video of showing the chip-bonding characteristic (3D bi-phasic conductor).
